# Supplementary material for: The Russo-Ukrainian War’s toll on paediatric health during the first two years and future research directions: a scoping review
Source: Commun Med (Lond). 2025 Oct 23;5:431. doi: 10.1038/s43856-025-01190-1 (PMC12549923; doi:10.1038/s43856-025-01190-1)
Supplement: Supplementary file 2 — Description of the additional supplementary file [file 43856_2025_1190_MOESM2_ESM.pdf]

**Description of additional supplementary file**

File name: Supplementary data
Description: The supplementary data file lists the final articles included in the study, t ype of article, as well as identified main and subtopics.
